# Depression, Anxiety, Resilience, and Family Functioning Among Different Age Groups During the COVID-19 Pandemic: A One-Year Longitudinal Study

**DOI:** 10.3390/healthcare13030237

**Published:** 2025-01-24

**Authors:** Vasiliki Efstathiou, Athanasia Papadopoulou, Valeria Pomini, Katerina Chatzimichail, Ioannis Michopoulos, Eleni Vousoura, Pilios-Dimitris Stavrou, Adamantia Kaparoudaki, Marianna Papadopoulou, Nikolaos Smyrnis, Athanasios Douzenis, Rossetos Gournellis

**Affiliations:** 1 Department of Psychology, School of Philosophy, National and Kapodistrian University of Athens, 15772 Athens, Greece; vefstathiou@psych.uoa.gr (V.E.); evousoura@psych.uoa.gr (E.V.); pstavrou@psych.uoa.gr (P.-D.S.); 2 Second Department of Psychiatry, School of Medicine, “Attikon” University General Hospital, National and Kapodistrian University of Athens, 12462 Athens, Greece; athanpapad@hotmail.gr (A.P.); imihopou@med.uoa.gr (I.M.); smyrnis@med.uoa.gr (N.S.); thandouz@med.uoa.gr (A.D.); 3 First Department of Psychiatry, School of Medicine, “Eginition” Hospital, National and Kapodistrian University of Athens, 11528 Athens, Greece; vpomini@med.uoa.gr; 4 Second Department of Radiology-Radiotherapy, School of Medicine, “Attikon” University General Hospital, National and Kapodistrian University of Athens, 12462 Athens, Greece; katerina@hcsl.com; 5 Hellenic Center of Mental Health and Research, 12243 Athens, Greece; akaparoudaki@ekepsye.gr; 6 Department of Physiotherapy, University of West Attica, 12243 Athens, Greece; mpapad@uniwa.gr

**Keywords:** mental health, trajectory, pandemic, prospective study, age

## Abstract

**Background/Objectives**: The COVID-19 pandemic has profoundly disrupted mental health globally, affecting individuals across all age groups. Understanding its long-term impact is crucial for identifying age-specific vulnerabilities and informing targeted mental health interventions. This longitudinal study aimed to investigate the within-person changes in mental health across different age groups in Greece from the first to the third pandemic wave of coronavirus disease 2019 (COVID-19) (i.e., one year later) during nationwide lockdowns. It further compared the mental health outcomes of three distinct age groups, stratified based on their vulnerability to COVID-19—younger adults (18–29 years), adults (30–59 years), and older adults (60–84 years)—and examined correlates of depression and anxiety during the third pandemic wave. **Methods**: A total of 720 participants—92 younger adults, 543 adults, and 85 older adults—completed the same set of questionnaires during the first (April–May 2020) and third (March–May 2021) pandemic-related lockdowns. At both time points, participants provided data on the Generalized Anxiety Disorder-2, Patient Health Questionnaire-2, Systemic Clinical Outcome and Routine Evaluation-15, Connor–Davidson Resilience Scale-2, and demographic information. **Results**: Results indicated a within-individual increase in depression and a decrease in resilience for all participants, irrespective of age. Anxiety increased only among younger adults and adults, whereas family functioning remained stable across all age groups one year post-pandemic onset. Furthermore, younger adults reported higher levels of depression and anxiety, lower resilience, and more impaired family functioning compared to the older age groups in both lockdowns. Among the different age groups, factors independently associated with depression and anxiety were identified through stepwise regression analyses. **Conclusions**: The present study provides evidence for mental health deterioration during the pandemic across all age groups, with younger adults exhibiting heightened vulnerability.

## 1. Introduction

The coronavirus disease 2019 (COVID-19) pandemic has profoundly impacted mental health globally. This unprecedented public health crisis presented not only a threat to physical health but also created widespread psychological distress due to significant disruptions to everyday life [1]. A recent review indicated that depression and anxiety have shown significant increases during the pandemic compared to pre-pandemic times, underscoring the multifaceted effect on mental health [2]. These increased levels of distress have been associated with multiple factors, including social isolation, economic uncertainty, fear of infection, and grief resulting from the loss of close relatives and friends.

The impact of the pandemic has differed considerably among adults with different characteristics, with particularly severe consequences for vulnerable populations such as older adults as they experienced more severe complications and higher mortality rates due to the disease [3]. Restriction measures, imposed to attenuate the viral spread, in turn affected the access to both physical and mental healthcare services [4,5]. A recent review showed that the use of mental health services declined at the onset of the pandemic and while its rates increased later in 2020 and through 2021, they remained lower than the pre-pandemic levels for certain services [1]. Furthermore, restrictions on religious gatherings disrupted an important source of social and emotional connection, compounding feelings of isolation and anxiety for many individuals, while older adults, who often rely on these gatherings for support, were likely particularly affected [6].

Despite initial concerns regarding the consequences of the COVID-19 pandemic on the mental health of older adults, research during the first months of the pandemic revealed that they demonstrated notable resilience against its adverse effects, suggesting that they may be less negatively affected compared to other age groups [4]. In contrast, a number of studies have highlighted the fact that younger individuals might be in a more vulnerable state as they experienced more pronounced mental health challenges than adults and older adults [7]. Of note, a longitudinal study conducted from 2020 to 2022 found that younger age was a significant risk factor for depressive symptoms regardless of sex [8].

However, there is evidence that while older adults were less psychologically distressed than younger adults, they still demonstrated an increase in depressive, anxiety, and stress symptoms, as well as loneliness *throughout the course* of the pandemic as compared to before [9,10,11]. Regarding depression among older individuals, women demonstrated a greater increase in response to the pandemic, despite experiencing similar changes in depression levels compared to men before the pandemic [12]. Factors such as internet use, high mastery, larger social networks, female gender, praying, and COVID-19 vaccination have been found to mitigate these adverse effects [10]. A number of studies have used longitudinal research designs to investigate changes in mental health during the COVID-19 pandemic; however, these are less prevalent compared to cross-sectional or repeated cross-sectional studies. Furthermore, these studies frequently compare psychological outcomes with pre-pandemic data, offering valuable insights but often overlooking how these factors evolve within the pandemic [1]. Existing research tends to focus on anxiety and depression, with less attention being directed toward positive constructs such as resilience and family functioning, while the long-term effects of the COVID-19 pandemic across different age groups remain underexplored.

To this end, this longitudinal study focuses on the following objectives:

(i) To investigate within-person changes in depression, anxiety, resilience, and family functioning across time, specifically from the first COVID-19 lockdown to the third lockdown, among younger adults (18–29 years), adults (30–59 years), and older adults (60+ years).
(ii) To compare the three age groups at both the first and third lockdowns regarding levels of depression, anxiety, resilience, and family functioning.
(iii) To identify socio-demographic and psychological correlates of depression and anxiety during the third lockdown and examine how these correlates differ across the three age groups.

## 2. Materials and Methods

### 2.1. Participants and Process

The methods of this study have been described in detail previously [13]. Briefly, participants were eligible for inclusion if they were adults (aged 18 years or older) who completed both surveys (i.e., baseline and follow-up). Individuals were excluded if they provided incomplete questionnaires or did not consent to participate in the follow-up study. During the first COVID-19 wave and while the first national pandemic-related lockdown was imposed (7 April to 3 May 2020), 5116 adults participated in an online survey aimed at assessing the social and psychological impact of the COVID-19 pandemic.

**Figure 1:**
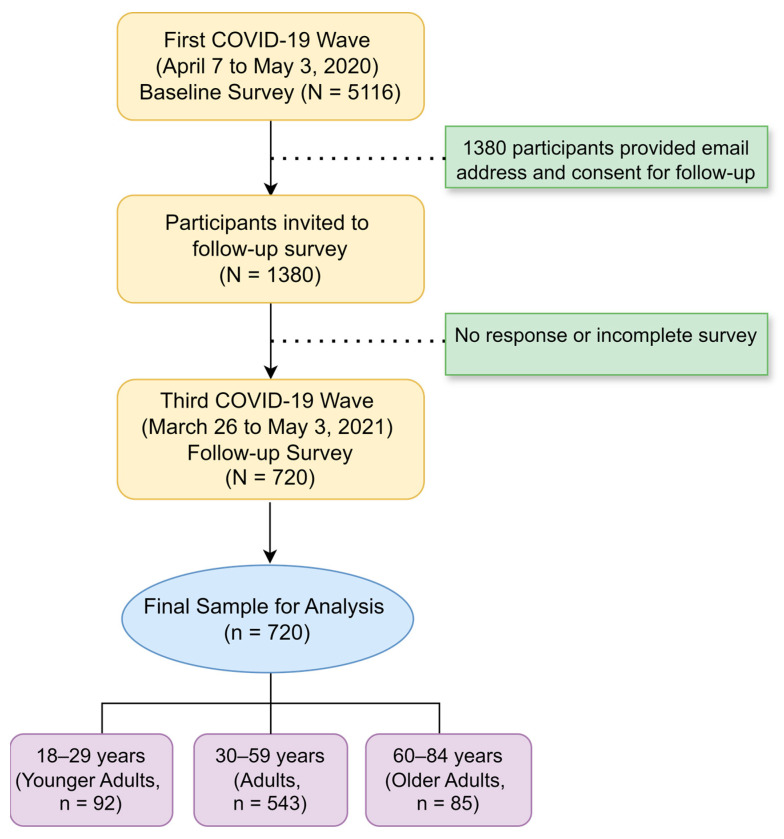
Flow diagram of participant recruitment and inclusion across COVID-19 survey waves.

Almost one year later, during the third wave of the pandemic while a third national lockdown was in effect (26 March to 3 May 2021), 1380 participants from the first survey, who had provided us with their emails for a follow-up survey, were invited to respond to the same questions. Of these 1380, a total of 720 adults completed the questionnaire (Figure 1).

In particular, the sample comprised 92 [12.8%; 67 (72.8%) females] younger adults aged between 18 and 29 years, 543 [75.4%; 410 (75.5%) females] adults aged between 30 and 59 years, and 85 [11.8%; 54 (63.5%) females] older adults aged between 60 and 84 years. Although developmental frameworks often classify young adulthood as extending beyond age 29, our age brackets were determined according to epidemiological data on COVID-19 outcomes [6]. According to that study, individuals aged below 30 and those above 59 showed distinctly different symptomatic case fatality risks compared to those aged 30–59 (0.6 [0.3–1.1] and 5.1 [4.2–6.1] times more likely to die, respectively). Consequently, 18–29 years and 60–84 years were grouped separately from the intermediate category of 30–59 years to reflect these heightened or lowered risks [14].

The study received approval from the Institutional Review Board of the University General Hospital “Attikon” of Athens and complied with the principles laid down in the declaration of Helsinki. Web-based informed consent was obtained from all participants. In particular, all individuals were informed before proceeding to the questionnaire form, regarding the purpose of the study and their voluntary participation. Furthermore, all were assured confidentiality, anonymity, and the right to withdraw at any time, without giving any reason and without consequences.

### 2.2. Measures

Respondents completed a questionnaire developed for the study’s purposes, as well as the following psychometric instruments: (i) Generalized Anxiety Disorder [15] (GAD-2) as a brief measure of anxiety; (ii) Patient Health Questionnaire [16] (PHQ-2) for the assessment of depression; (iii) Systemic Clinical Outcome and Routine Evaluation [17] (SCORE-15) to determine family functioning levels; and (iv) Connor–Davidson Resilience Scale [18] (CD-RISC-2) as a brief measure of resilience. A lower total score in SCORE-15 was indicative of higher family functioning, whereas a higher total score in CD-RISC-2 represented higher resilience. Cronbach’s alpha values for the questionnaires used in the study were as follows: GAD-2 (α = 0.85), PHQ-2 (α = 0.82), SCORE-15 (α = 0.89), and CD-RISC-2 (α = 0.80).

### 2.3. Statistical Analysis

Two-way mixed analyses of variance (ANOVA) were performed in order to assess the within-individual changes on mental health variables over time (i.e., from the first lockdown to the third, one year later), and differences between the three age groups (i.e., younger adults 18 to 29 years of age, adults 30 to 59 years of age, and older adults 60 to 84 years of age) during the first and third lockdown period.

Multiple linear regression analyses were conducted to explore socio-demographic and psychological correlates of depression and anxiety during the third lockdown, with separate models applied for each age group (younger adults, adults, and older adults) to account for potential differences across cohorts. Conducting a single pooled analysis could obscure age-specific variations in predictors; therefore, separate models allowed for a more nuanced examination of each subgroup. A stepwise selection procedure was used to determine statistically significant correlates among the following variables: age, education, marital status, living alone, having children, being vulnerable for COVID-19 severe infection due to frail health, worry regarding COVID-19 infection, living with a person vulnerable for COVID-19 severe infection, sleep quality post-COVID-19 outbreak, positive feelings regarding the lockdown measures, self-reported coping strategies during the pandemic (i.e., use of personal skills, relationship with family, relationship with friends, faith in a Supreme Being), family functioning, resilience, anxiety, and depression. The first survey’s depression and anxiety variables were also included in the respective models, while gender was used as a control variable in all models and was therefore retained despite being statistically non-significant. Statistical analyses were performed using IBM SPSS Statistics for Windows, Version 29.0 (IBM Corp., Armonk, NY, USA).

## 3. Results

The two-way mixed ANOVA analyses’ findings are presented in Table 1.

As regards depression, a within-individual increase from the first lockdown to the third lockdown was revealed for all age groups, with younger adults presenting higher levels than adults of the older age groups (i.e., 30 to 59 and 60 and above) at both points in time, while no interaction was found.

Regarding resilience, a within-individual decrease from the first lockdown to the third lockdown emerged for all age groups. In addition, at both points in time, younger adults presented lower levels than individuals of the older age groups (i.e., adults and older adults), while no interaction was found.

Family functioning did not alter significantly between the two lockdowns for any of the age groups, while no interaction was found. As concerns family functioning at both lockdowns, younger adults presented more impaired levels of family functioning compared to adults of the other age groups

Regarding anxiety, a statistically significant interaction of time and age emerged. A within-individual age group increase from the first lockdown to the third lockdown was revealed among younger adults and adults, while no change was found among older adults. At both points in time, younger adults presented higher levels of anxiety than both older age groups.

Table 2 presents the stepwise regression analyses for depression in each age group, highlighting the factors that significantly predicted depressive levels in the third lockdown.

Higher depression during the first wave predicted higher depression levels in the third wave among both the younger adult and adult groups. Across all age groups, higher anxiety was independently associated with higher depression levels. Worsened sleep quality was independently related to higher depression in the adult and older adult groups. Furthermore, regarding the younger adult group, protective factors for depression included female gender, faith in a Supreme Being, and reliance on personal skills as coping strategies. In the adult group (30 to 59 y.), protective factors included positive feelings towards the lockdown measures and reliance on personal skills as a coping strategy. Finally, in the older adult group, protective factors for depression included higher resilience and lower levels of worry about COVID-19 infection.

Table 3 presents the stepwise regression analyses for depression in each age group, indicating the factors that significantly predicted anxiety levels in the third lockdown.

Higher anxiety levels during the first pandemic wave predicted higher anxiety levels during the third wave for the two older age groups (30 to 59 y. and 60 to 84 y.), but not within the younger adult group. Third wave depression levels were independently associated with third wave anxiety levels among all age groups, while worsened sleep quality was related to higher anxiety levels only in the adult group.

In terms of protective factors for anxiety levels during the third wave, these included male gender in the younger adult group; higher family functioning, resilience, and having children among the adult group; and having children and positive feelings toward the lockdown measures in the older adult group.

## 4. Discussion

This longitudinal study investigated within-individual age group changes in depression, anxiety, resilience, and family functioning from the first wave of the COVID-19 pandemic to the third wave in a sample of 720 participants aged 18–84 years, while nationwide lockdowns were in effect. It also examined factors independently associated with depression and anxiety across the different age groups during the third wave.

A significant increase in depression and a significant decrease in resilience emerged for all individuals regardless of their age, whereas anxiety increased only among younger adults (18 to 29 y.) and adults (30 to 59 y.), but not in older adults (60 to 84 y.) Family functioning remained unaltered one year post-pandemic onset across all age groups. To the best of our knowledge, only a few studies [19,20,21] have prospectively investigated the long-term psychological effects of the pandemic’s different waves on older adults. However, in these prospective studies, the psychosocial effects of the pandemic have not been assessed comparatively across different age groups. In contrast with the results of the current study, which showed that depression increased and anxiety remained stable among older adults (60 years and older), a Spanish longitudinal study on older adults’ mental health during the pandemic (i.e., April 2020, July 2020, and January 2021) demonstrated that depression remained stable, while anxiety significantly decreased [19]. In the same study, resilience and family functioning were also measured, but while means ± SDs are provided for each time point, differences have not been statistically computed. However, according to the observed values, family functioning appears stable over time, while there is a trend for a decrease in resilience from April to July 2020. Nevertheless, there is a paucity of studies investigating the mental health of older adults prospectively over the course of different pandemic waves. Several studies investigate differences with pre-pandemic levels or explore pandemic waves only during 2020; in particular, a longitudinal study of older adults in Chile demonstrated that as compared to before the pandemic onset, older adults during the pandemic (June to September 2020) presented higher depressive and anxiety symptoms, but also higher resilience [20]. Additionally, another longitudinal cohort study, with a sample consisting of older adults that lived in England, showed that depression, loneliness, and poor life quality increased significantly during June/July 2020 compared with pre-pandemic levels and continued to worsen during the second national lockdown in November/December 2020, with further increases in anxiety symptoms from June/July 2020 to November/December 2020 [21].

In this study, investigating possible changes one year post-pandemic onset, younger adults (18 to 29 y.) presented more impaired levels of depression, anxiety, resilience, and family functioning compared to adults (30 to 59 y.) and older adults (60 to 84 y.) during both lockdowns. This is in line with other studies conducted during the pandemic, which reported that older adults displayed comparatively lower levels of depression and anxiety [7,22,23]. Crucially, a recent WHO review on the mental health pandemic impact suggested increases in major depressive disorder and anxiety disorder, indicating that younger adults were more affected than older individuals [24].

Furthermore, this study investigated factors that were independently associated with depression and anxiety among different age groups during the third pandemic wave. Higher depression during the first wave predicted higher depression in the third wave among younger adults and adults, but not in the older adult group, whereas higher anxiety in the first wave predicted anxiety during the third wave among the adult and older adult groups, but not within the younger adult group. Furthermore, among the older adult group, depression correlates during the third wave included anxiety, worsened sleep quality, and COVID-19 infection worry. Anxiety correlates in the older adult group included, apart from anxiety during the first wave, depression during the third wave; protective factors included having children and positive feelings for the lockdown measures. Having an intimate friend network has been identified as a significant factor for enhancing life satisfaction among older adults, underlining the importance of social connections in mitigating the impacts of social isolation experienced during the pandemic [25]. A systematic review exploring factors associated with depression and anxiety in older adults during the pandemic suggested that female gender, loneliness, poor sleep quality, and poor motor function were associated with both depression and anxiety, while having a stable and high monthly income related with lower depression and anxiety, and exercising was related to lower depression [26]. In the current study, gender was independently associated with depression (men presented higher levels) and anxiety (women presented higher levels) only among the younger adult group.

## 5. Strengths and Limitations

This study has several strengths. It employs a longitudinal design, allowing for the assessment of within-individual changes in mental health variables over a one-year period during the COVID-19 pandemic. The inclusion of a large sample spanning three age groups allows for meaningful comparisons across the lifespan. Additionally, the use of validated psychometric instruments ensures reliable measurements.

Despite its strengths, the study also has limitations that should be acknowledged. The sample was recruited online, which may have introduced selection bias, as individuals with limited internet access or technological literacy may have been underrepresented. Additionally, the study relied on self-reported measures, which, while efficient for large-scale data collection, are susceptible to response and recall biases. Furthermore, although including a large sample can enhance statistical power, it can also detect very small differences as being statistically significant. In our study, the differences observed were consistent across multiple outcomes, which suggests they may reflect genuinely meaningful changes rather than mere artifacts of an overpowered sample. However, we acknowledge that calculating formal effect sizes would provide additional clarity regarding the clinical significance of these findings. Finally, a formal power analysis was not conducted prior to data collection; therefore, the study’s sample size was determined by the number of participants who agreed to complete both the baseline and follow-up surveys rather than by an a priori calculation of expected effect sizes. While this approach allowed for a more naturalistic sampling during the rapidly evolving COVID-19 pandemic, it may limit the generalizability of certain findings.

## 6. Conclusions

This study provides evidence that the COVID-19 pandemic negatively impacted mental health across different age groups over time. Specifically, younger adults (18–29) exhibited consistently higher levels of depression and anxiety, whereas older adults (60–84) showed a notable increase in depression and a decrease in resilience from the first to the third pandemic wave. These findings underline the urgent need for age-specific mental health support and interventions.

Clinically, improving access to mental healthcare for older adults is crucial, given their increased vulnerability, yet our results highlight that younger adults also require targeted strategies to address their higher levels of anxiety and depression. Furthermore, the association of third-wave depression (among younger adults and adults) and anxiety (among adults and older adults) with baseline symptoms emphasizes the importance of early identification and intervention—ideally implemented in the initial stages of a crisis. Such age-focused interventions might include targeted screening for sleep disturbances, family dysfunction, and COVID-19-related worries, as well as resilience-building programs tailored to each group’s most pressing needs.

In addition, the need for mental health services to lower fears of infection, manage insomnia problems, support family function, and enhance positive attitudes towards preventive measures should also not be overlooked. The within-individual increase in depression and the decrease in resilience observed in all age groups constitute a warning, highlighting the need for continued follow-up studies to track long-term psychopathological trends and refine effective, age-tailored mental health strategies.

## Figures and Tables

**Table 1:** Within-person changes from the first to the third pandemic wave/lockdown in Greece by age categories.

	Pandemic Wave/Lockdown			Age Categories				
	First	Third	Time Effect F/*p*	Younger Adults (18–29 y.)	Adults (30–59 y.)	Older Adults (60–84 y.)	Age Effect F/*p*	Time × Age F/*p*
**Depression**	1.46 (1.63)	1.91 (1.70)	**28.82/<0.001**	2.13 (1.83) ^a^	1.62 (1.64) ^b^	1.62 (1.70) ^b^	**5.26/0.005**	1.62/0.314
**Resilience**	6.25 (1.47)	6.04 (1.47)	**4.13/0.042**	5.70 (1.58) ^a^	6.21 (1.43) ^b^	6.22 (1.57) ^b^	**5.99/0.003**	1.31/0.271
**Family functioning**	28.22 (10.36)	28.42 (10.53)	0.65/0.421	34.06 (12.28) ^a^	27.41 (9.92) ^b^	27.80 (9.57) ^b^	**18.99/** **<0.001**	2.10/0.123
**Anxiety** ^§^	1.10 (1.40)	1.44 (1.50)	**25.20/** **<0.001**	1.46 (1.56)	1.06 (1.29)	0.94 (1.36)	**11.43/** **<0.001**	**3.52/0.030**
**Anxiety in younger adults (18–29 y.)**	1.46 (1.57) ^a,‡^	2.15 (1.83) ^b,‡^						
**Anxiety in adults (30–59 y.)**	1.06 (1.30) ^a,†^	1.38 (1.45) ^b,†^						
**Anxiety in older adults (60 to 84 y.)**	0.94 (1.37) ^†^	1.08 (1.19) ^†^						

Notes: Descriptive statistics presented as mean (standard deviation). Means (SD) with differing superscripts within columns (^a^, ^b^) and rows (^†^, ^‡^) are statistically significantly different (*p* < 0.001). Bonferroni correction applied for multiple comparisons, when appropriate. ^§^ Due to a significant interaction of Time × Age on anxiety, simple main effects were calculated and subcategories’ descriptives are also reported. Time effect d.f. = 1, 717; age categories effect d.f. = 2, 717; interaction d.f. = 2, 717. bold style indicates the statistically significant.

**Table 2:** Stepwise multiple linear regression analyses for depression levels during the third COVID-19 pandemic wave/lockdown in Greece.

Younger Adults (18–29 y.)			Adults (30–59 y.)			Older Adults (60–84 y.)		
Model: F (5, 84) = 30.41 *p* < 0.001, adj. R^2^ = 0.623			Model: F (7, 535) = 51.33 *p* < 0.001, adj. R^2^ = 0.394			Model: F (6, 76) = 37.66, *p* < 0.001, adj. R^2^ = 0.728		
Variables	Coef. b	95% CI	Variables	Coef. b	95% CI	Variables	Coef. b	95% CI
**Depression during the first wave** (per unit)	0.28 ***	0.13 to 0.44	**Depression during the first wave** ca(per unit)	0.20 ***	0.12 to 0.27	**Resilience** (per unit)	−0.17 *	−0.32 to −0.03
**Anxiety** (per unit)	0.57 ***	0.42 to 0.72	Anxiety (per unit)	0.51 ***	0.43 to 0.60	**Anxiety** (per unit)	0.95 ***	0.76 to 1.13
**Personal skills as a coping strategy during the pandemic**	−0.93 **	−1.51 to −0.35	**Sleep quality after COVID-19 outbreak**, Worsened/somewhat worsened vs. Remained the same	0.31 *	0.07 to 0.55	**Sleep quality after COVID-19 outbreak**, Worsened/somewhat worsened vs. Remained the same	0.76 **	0.34 to 1.19
**Faith in a Supreme Being as a coping strategy during the pandemic**	−0.97 *	−1.72 to −0.22	**Sleep quality after COVID-19 outbreak**, Improved/somewhat improved vs. Remained the same	0.18	−0.24 to 0.57	**Sleep quality after COVID-19 outbreak**, Improved/somewhat improved vs. Remained the same	−0.93	−1.88 to 0.02
**Gender**, Women vs. Men	−0.61 *	−1.18 to −0.03	**Personal skills as a coping strategy during the pandemic**	−0.37 **	−0.63 to −0.10	**Worry regarding COVID-19 infection** (per unit)	0.13 *	0.04 to 0.22
			**Positive feelings regarding the lockdown measures**, Yes vs. No	−0.27 *	−0.52 to −0.02	**Gender**, Women vs. Men	0.01	−0.42 to 0.44
			**Gender**, Women vs. Men	0.25	−0.01 to 0.51			

**Notes**: A 95% confidence interval: 95% CI; * *p* < 0.05, ** *p* < 0.01, *** *p* < 0.001. Except for the variables designated as first wave factors, all other variables originate from the third COVID-19 pandemic wave.

**Table 3:** Stepwise multiple linear regression analyses for anxiety levels during the third COVID-19 pandemic wave/lockdown in Greece.

Younger Adults (18–29 y.)			Adults (30–59 y.)			Older Adults (60–84 y.)		
Model: F (2, 87) = 48.29, *p* < 0.001, adj. R^2^ = 0.515			Model: F(8, 534) = 64.17, *p* < 0.001, adj. R^2^ = 0.483			Model: F (5, 79) = 35.68, *p* < 0.001, adj. R^2^ = 0.674		
Variables	Coef. b	95% CI	Variables	Coef. b	95% CI	Variables		
**Depression** (per unit)	0.69 ***	0.55 to 0.83	**Anxiety during the first wave** (per unit)	0.25 ***	0.17 to 0.32	**Anxiety during the first wave** (per unit)	0.14 *	0.01 to 0.27
**Gender**, Women vs. Men	0.83 **	0.21 to 1.45	**Depression** (per unit)	0.35 ***	0.29 to 0.41	**Depression** (per unit)	0.46 ***	0.35 to 0.57
			**Children**, Yes vs. No	−0.22 *	−0.41 to −0.03	**Children**, Yes vs. No	−0.66 **	−1.07 to −0.24
			**Sleep quality after COVID-19 outbreak**, Worsened/somewhat worsened vs. Remained the same	0.32 **	0.13 to 0.52	**Positive feelings regarding the lockdown measures**, Yes vs. No	−0.34 *	−0.65 to −0.03
			**Sleep quality after COVID-19 outbreak**, Improved/somewhat improved vs. Remained the same	−0.23	−0.56 to 0.11	**Gender**, Women vs. Men	−0.02	−0.33 to 0.29
			**Family dysfunction** (per unit)	0.02 *	0.01 to 0.03			
			**Resilience** (per unit)	−0.15 ***	−0.22 to −0.09			
			**Gender**, Women vs. Men	0.18	−0.03 to 0.38			

**Notes**: A 95% confidence interval: 95% CI; * *p* < 0.05, ** *p* < 0.01, *** *p* < 0.001. Except for the variables designated as first wave factors, all other variables originate from the third COVID-19 pandemic wave.

## Data Availability

The data presented in this study are available on reasonable request from the corresponding author.
